# Predictors of euthyreosis in hyperthyroid patients treated with radioiodine ^131^I^−^: a retrospective study

**DOI:** 10.1186/s12902-020-00551-2

**Published:** 2020-06-01

**Authors:** Albert Stachura, Tomasz Gryn, Bernadetta Kałuża, Tadeusz Budlewski, Edward Franek

**Affiliations:** 1 Department of Internal Medicine, Endocrinology and Diabetology, Central Clinical Hospital of the Ministry of the Interior, Wołoska 137, 02-507 Warsaw, Poland; 2 Nuclear Medicine Department, Central Clinical Hospital of the Ministry of the Interior, Warsaw, Poland; 3 Department of Human Epigenetics, Mossakowski Medical Research Centre, Polish Academy of Sciences, Warsaw, Poland

**Keywords:** Radioiodine, Hyperthyroidism, Euthyreosis, Graves’ disease, Toxic multinodular goiter, Predictors, Factors, 131I^−^

## Abstract

**Background:**

Radioiodine (RAI) treatment for hyperthyroidism is a very common modality, chosen by physicians worldwide. The outcome of the therapy, however, is not always predictable. While rendering a patient hypo- or euthyroid is meant as a therapeutic success, the latter does not require lifelong hormonal supplementation. The aim of our study is to determine predictors of euthyreosis in patients who underwent RAI treatment.

**Methods:**

Medical records of 144 patients who had undergone RAI therapy were examined. Laboratory and clinical data were analyzed statistically. Ultrasonography findings, such as thyroid volume, nodules’ size and characteristics had been collected at the beginning of treatment and 6 months after the administration of radioiodine ^131^I^−^. Moreover, scintigraphy results were taken into account. Multivariate logistic regression analysis model has been used to find predictors of euthyroidism after 12 months of follow-up. The predictors of normal thyroid function have also been analyzed separately for patients with GD (Graves’ disease) and TMNG (toxic multinodular goiter).

**Results:**

The analysis showed that age (OR 1,06; 95%CI 1.025-1.096, *p* = 0,001), thyroid gland volume (OR 1,04; 95%CI 1,02-1,06; *p* < 0.001) and iodine uptake level (OR 0,952; 95%CI 0,91-0,98; *p* = 0,004) were significant factors of achieving normal thyroid function after RAI therapy. According to multivariate logistic regression analysis, in GD patients only age has been shown to be a significant factor (OR 1,06; 95%CI 1,001-1,13; *p* = 0.047), while in TMNG patients’ age (OR 1,04; 95%CI 1–1,09; *p* = 0.048), thyroid gland volume (OR 1.038; 95%CI 1.009-1.068; *p* = 0.009) and iodine uptake level (OR 0.95; 95%CI 0.9–0.99; *p* = 0.02) all have been proven to be significant predictors of achieving euthyroidism.

**Conclusions:**

The more advanced age, larger volume of thyroid gland and lower iodine uptake level are predictors of euthyreosis after RAI treatment.

## Background

**Fig. 1:**
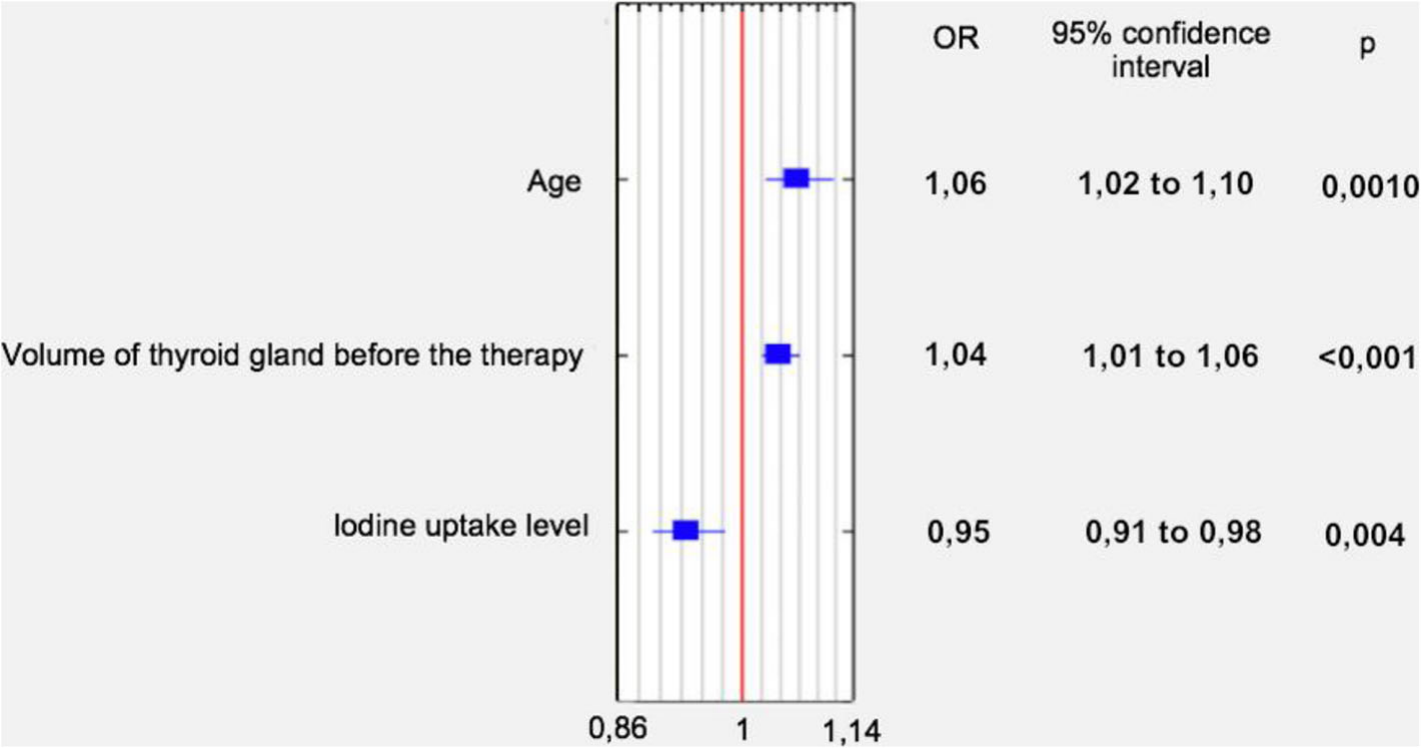
Factors contributing to euthyreosis 12 months after RAI administration. Multivariate logistic regression analysis showed three predictors in the euthyroid outcome group [*n* = 52]

Hyperthyroidism is one of the most prevalent thyroid disorders, affecting 0.5–2% of women (tenfold more than man) in iodine-replete environments [1]. The most common causes of this condition are Graves’ disease (GD), toxic multinodular goiter (TMNG) and thyroid adenoma (TA) [1, 2]. Currently, there are three possible approaches to treatment of hyperthyroidism: antithyroid drugs (ATDs), radioiodine (RAI) therapy, and thyroidectomy. In case of GD, RAI therapy remains the most popular choice of the physicians in the United States, while ATDs are preferred in Europe, Latin America and Japan [3, 4]. RAI therapy is increasingly used as a first-line treatment of hyperthyroidism as data accumulates proving its efficacy and safety [5, 6]. According to the American Society of Endocrinology, radioiodine treatment may be used in case of all three major causes of hyperthyroidism, depending on the disease’s progression [7]. The outcome of RAI therapy, however, is difficult to predict. An optimal dose that would both eliminate the risk of developing hypothyroidism and maximize the chance of curing hyperthyroidism is individual and difficult to determine [8] (Fig. 1).

The success of RAI therapy in hyperthyroidism – meant as rendering the patient hypo- or euthyroid - correlates strongly with the activity of administered radioiodine [7]. There are several studies comparing the efficacy of treatment using a fixed dose versus a calculated dose, showing little differences between the two approaches [9–14]. Thus, the choice of the method depends solely on the physician [7]. Undoubtedly, the outcome of the RAI therapy is not fully predictable and may result in hypothyroidism, euthyroidism or unchanged hyperthyroidism [7]. In an attempt to determine possible factors that could influence the outcome of the treatment, several studies have been conducted, proving e.g. gender, age, presence of ophthalmopathy and serum free T3 concentration to be relevant [11–13, 15–18].

Radioiodine therapy is the only non-operative treatment modality that provides a possibility of rendering a patient euthyroid without the necessity of further pharmacological management. There are, however, not much data on the predictors of achieving euthyreosis in the patients after RAI therapy, presence of which may facilitate the choice of treatment [19–21]. Thus, in our research we aim to find predictors of specifically euthyreosis in patients who underwent the RAI treatment.

## Methods

### Patients

In our study, we included 144 patients transferred for RAI treatment at the Nuclear Medicine Department of the Central Clinical Hospital of the Ministry of the Interior and Administration in Warsaw. All of them were diagnosed with either GD or TMNG. Data regarding the patients with TMNG were collected from the years 2015 and 2016, whereas in case of GD patients, we included data from years 2013–2016. Subjects, who had undergone 2nd RAI treatment, were also included. Thyroid ultrasound examination was performed before radioiodine administration and 6 months after the procedure. Following factors were defined and compared: volume of the thyroid gland, presence and dimensions of nodules. Moreover, results of scintigraphy were taken into consideration: iodine uptake, distribution, type of nodules (‘hot’ or ‘cold’). All the drugs proven to affect the activity of the thyroid gland (e.g. beta-blockers, amiodarone, antithyroid drugs), if taken by the patient, were also noted.

The fixed doses of ^131^I^−^ administered to patients varied between 610 and 790 MBq. All antithyroid drugs, if given, were withdrawn 1 week before radioiodine administration. Patients were subsequently observed for 12 months. Before the procedure, as well as after 2 weeks and 1, 2 and 6 months the serum concentrations of TSH, free T3 and free T4 were measured. Information regarding thyrostatic drugs or hormonal replacement therapy given during the observation period - in order to achieve euthyreosis - was also recorded. After 12 months, patients were divided into three groups: (1) patients with hypothyroidism, (2) patients who achieved euthyreosis (without medication), (3) patients with persistent hyperthyroidism. Patients were also divided into two etiological subgroups: subjects with Graves’ disease and with toxic multinodular goiter. Euthyreosis was meant as the serum concentrations of free T4, free T3 and TSH in the range of laboratory norms. RAI therapy rendered hypothyroidism if after the 12-month observation period hormonal replacement therapy was needed to achieve normal levels of thyroid hormones. On the other hand, hyperthyroidism was meant as the free T4 and T3 serum concentrations elevated or normal and TSH level decreased, which required antithyroid drugs’ administration.

We performed both univariate and multivariate logistic regression analysis for factors such as age, gender, thyroid volume, ATD treatment 2 weeks before iodine administration, fT3 concentration at presentation and iodine uptake. Predictors of achieving normal thyroid function were searched in three groups: (1) all the patients, (2) GD patients, (3) TMNG patients.

We also made follow-up calls to gather data on the patients’ thyroid status 12 months after radioiodine administration. Among the contacted patients, only in 8 the thyroid status changed between the 6th and 12th month post-therapy. In 6 cases patients previously rendered euthyroid, turned hypothyroid, whereas in 2 persistently hyperthyroid subjects, a normal thyroid function was achieved, without the 2nd dose of radioiodine.

### Statistical analysis

We used the program Statistica 13 (StatSoft Polska LTD) for the statistical analysis. The *p*-value below 0.05 was assumed as statistically significant. In order to determine the statistical significance, we used various tests according to the distribution of variables and their characteristics (Student’s t-test, Mann Whitney U test, chi-square test, Kruskal-Wallis ANOVA test). ROC curves were used for optimization of the threshold setting for selected predictors. We also used the logistic regression, Cox proportional hazards model analysis (12-months observation) to determine independent predictors of achieving normal thyroid function.

## Results

All the important clinical data are presented in Table 1. After 12 months of follow-up, 81 patients were rendered hypothyroid (group 1), requiring further hormonal supplementation, 52 achieved euthyreosis (group 2) and 11 remained in the state of hyperthyroidism (group 3). There were 98 females and 46 males in the studied group. Majority of the patients (eighty-one) were diagnosed with toxic nodular goiter and sixty-three with Graves’ disease. Among the patients, who achieved normal thyroid function after the therapy, only 8 were diagnosed with GD. Age of patients varied significantly from 53.5 ± 16.3 and 50.9 ± 16.4 in hypo- and hyperthyroid groups respectively to 67.3 ± 12.7 among subjects who achieved normal thyroid function. Among the patients who underwent thyroidectomy in the past, none became euthyroid after the treatment.

**Table 1:** Clinical information about the patients

	Group 1 (*n* = 81)	Group 2 (*n* = 52)	Group 3 (*n* = 11)	*p*-value	GD patients (*n* = 63)	TMNG patients (*n* = 81)	*p*
Males [n, %]	21(25.9)	21 (40.4)	4 (36.4)	0.2	16 (25.4)	30 (37)	0.23
Age [years]	53.5 ± 16.3	67.3 ± 12.7	50.9 ± 16.4	0.00001	48.6 ± 15.5	65.8 ± 12.9	0.000001
Graves’ disease [n, %]	48 (59.3)	8 (15.4)	7 (63.6)	0.000001			
Thyroidectomy in history [n, %]	6 (7.4)	0 (0)	1 (9.1)	0.12	2 (3.2)	5 (6.2)	0.41
Thyrostatic drugs used before RAI treatment [n, %]	20 (24.7)	15 (28.8)	6 (54.5)	0.12	13 (20.6)	28 (34.6)	0.06
Beta-blockers used before RAI treatment [n, %]	19 (23.4)	19 (36.5)	2 (18.2)	0.19	11 (17.5)	29 (35.8)	0.05
Steroids used before RAI treatment [n, %]	3 (3.7)	2 (3.8)	0 (0)	0.8	4 (6.3)	1 (1.2)	0.09
Orbitopathy [n, %]	1 (1.2)	0 (0)	0 (0)	0.67	1 (1.6)	0 (0)	0.26
Thyroid volume before RAI treatment [cm^3^]	22.6 ± 13	41.9 ± 22.2	54 ± 31.7	0.00001	24.3 ± 19.6	37.8 ± 21.3	0.000004
Thyroid volume 6 months after RAI treatment [cm^3^]	8.4 ± 12.6	23 ± 16.4	29.7 ± 20.1	0.00001	7.5 ± 9.1	21.4 ± 18.6	0.000001
Diffused nodules [n, %]	39 (54.9)	44 (84.6)	5 (50)	0.001	N/A		
‘Cold’ nodules	1 (1.2)	2 (3.8)	1 (9)	0.28	1 (1.6)	3 (3.7)	0.44
Iodine uptake [%]	36.4 ± 13.4	30.2 ± 11.3	54.3 ± 16.6	0.00001	40.1 ± 14.3	32.2 ± 13.4	0.0009
Uneven iodine distribution [n, %]	18 (22.2)	26 (50)	4 (36.4)	0.004	4 (6.3)	44 (54.3)	0.000001
Inferior poles of the thyroid lobes reaching jugular incision [n, %]	3 (3.7)	10 (19.2)	3 (27.3)	0.006	3 (4.8)	13 (16)	0.03
^131^I^−^ dose [MBq]	638.5 ± 44.2	658.1 ± 46.4	657.8 ± 64.8	0.035	624 ± 34.9	664.9 ± 48.4	0.00001
TSH concentration at presentation [μIU/ml]	1.5 ± 2.6	0.9 ± 1.7	0.6 ± 1	0.04	1.46 ± 2.9	1.08 ± 1.54	0.79
fT3 concentration at presentation [pg/ml]	4.1 ± 1.3	3.9 ± 1.6	5.6 ± 4.3	0.48	4.2 ± 1.9	4 ± 1.6	0.39
fT4 concentration at presentation [ng/ml]	5.5 ± 7.1	3.7 ± 5.1	2.8 ± 4.2	0.44	5.4 ± 6.8	4.1 ± 5.9	0.49
TSH concentration 6 months after RAI treatment [μIU/ml]	4.3 ± 5.7	2.3 ± 3.6	0.2 ± 0.25	0.00001	3.9 ± 6.5	2.7 ± 3.3	0.1
fT3 concentration 6 months after RAI treatment [pg/ml]	3.5 ± 1.1	3.9 ± 1.8	4.6 ± 2.3	0.79	3.8 ± 1.7	3.9 ± 0.9	0.65
fT4 concentration 6 months after RAI treatment [ng/ml]	3.4 ± 6.2	2.2 ± 3.3	1.9 ± 2	0.01	3.7 ± 6.6	2.1 ± 3.4	0.03

Patients have been assigned to the groups, based on the outcome of the RAI therapy after 12 months of follow-up. Group 1 – hypothyroid, group 2 – euthyroid, group 3 – hyperthyroid. Patients with GD and TMNG have been compared. *N/A* not applicable

### Ultrasonography examination and laboratory tests

All the data regarding ultrasonography and scintigraphy findings refer to the patients’ results before the RAI treatment, unless otherwise indicated. The effect of the therapy was strongly connected with the volume of the thyroid gland before RAI administration (*p* < 0.001). While patients with smaller initial thyroid volume (22.6 ± 13 cm3) were more prone to entering the state of hypothyroidism after the procedure, the larger thyroid glands (54 ± 31.7 cm3) predisposed to persistent hyperthyroidism. Among patients who had been rendered euthyroid, medium values (41.9 ± 22.2 cm3) of the thyroid glands were observed. Naturally, thyroid glands’ volumes were significantly larger in the TMNG group than in GD patients (*p* < 0.001).

Similarly, an association was found between the outcome of treatment and the size of thyroid gland 6 months after therapy (*p* < 0.001). Smaller volumes of thyroid gland (8.4 ± 12.6 cm3) were typical for patients, who presented with hypothyroidism, medium-sized thyroids (23 ± 16.4 cm3) for normal hormonal function and the largest volumes of the gland (29.7 ± 20.1 cm3) were characteristic for persistent hyperthyroidism.

Diffused nodules, found during ultrasound examination, occurred much more often in the patients from the 2nd group (84,6%) compared to individuals who entered the state of hypothyroidism (54,9%) or hyperthyroidism (50%) after treatment. On the other hand, presence of thyroid lobes reaching jugular incision was one of the factors contributing to failure of the therapy. Thyroid masses at the level of the thoracic inlet were present in 27,3% of patients with post-therapeutic hyperthyroidism, 19,2% patients with euthyroidism and only 3,7% with hypothyroidism.

An interesting relationship between the iodine uptake level and the outcome of the therapy was found. Patients with lower iodine uptake (30.2 ± 11.3%) had a significantly greater (*p* < 0.001) chance of entering the state of euthyroidism. Both remaining outcome groups had clearly higher iodine uptake levels (36.4 ± 13.4% for hypothyroidism and 54.3 ± 16.6% for hyperthyroidism). Patients with GD showed significantly higher iodine uptake levels (40.1 ± 14.3%) than patients with TMNG (32.2 ± 13.4%) (*p* < 0.001).

Among laboratory thyroid function tests, only TSH concentration at the beginning of the therapy seemed to be a significant factor contributing to predicting the outcome of therapy. Patients with lower concentrations (0.6 ± 1) were more prone to persisting hyperthyroidism (*p* = 0.035), medium concentrations (0.9 ± 1.7) pointed to normal hormonal function, while the highest values usually resulted in hypothyroidism (1.5 ± 2.6). There were no significant differences in TSH concentrations between GD and TMNG groups.

Results of uni- and multivariate logistic regression analyses are presented in Tables 2, 3 and 4. According to multivariate logistic regression analysis, there are three factors, contributing to achieving normal hormonal level 12 months after the therapy – age (OR 1.06; 95% CI 1.025–1.096; *p* = 0.001), thyroid volume (OR 1.04; 95% CI 1.02–1.06; *p* < 0.001) and iodine uptake (OR 0.95; 95% CI 0.91–0.98; *p* = 0.004). Similar results occurred in case of TMNG patients. Older patients (OR 1.04; 95% CI 1–1.09; *p* = 0.048), with larger thyroid glands (OR 1.038; 95% CI 1.009–1.068; *p* = 0.009) and lower iodine uptake levels (OR 0.95; 95% CI 0.9–0.99; *p* = 0.02) had bigger chances of achieving an euthyroid state. This is, however, not a case in GD patients, in whom only higher age was proven a significant factor of normal thyroid function (OR 1.06; 95% CI 1.001–1.13; *p* = 0.047).

**Table 2:** Factors contributing to achieving euthyreosis in all the patients treated with RAI

	Univariate logistic regression analysis			Multivariate logistic regression		
	Odds ratio	95% OR confidence interval	*p*-value	Odds ratio	95% OR confidence interval	*p*-value
Age [years]	1.07	1.04–1.09	0.00001	1.06	1.025–1.096	0.001
Gender [men]			> 0.05			> 0.05
Thyroid volume [ml]	1.04	1.02–1.06	0.0002	1.04	1.02–1.06	0.0001
Tyrostatic drugs taken 2 weeks before therapy	1.03	0.5–2.2	0.94			> 0.05
fT3 concentration at presentation	0.69	0.5–1.01	0.07			> 0.05
Iodine uptake [%]	0.952	0.92–0.98	0.001	0.95	0.91–0.98	0.004

**Table 3:** Factors contributing to achieving euthyreosis in patients with GD treated with RAI

	Univariate logistic regression analysis			Multivariate logistic regression		
	Odds ratio	95% OR confidence interval	*p*-value	Odds ratio	95% OR confidence interval	*p*-value
Age [years]	1.065	1.01–1.13	0.02	1.06	1.001–1.13	0.047
Gender [men]	1.94	0.4–9.2	0.4			> 0.05
Thyroid volume [ml]	1.03	0.99–1.06	0.09			> 0.05
Tyrostatic drugs taken 2 weeks before therapy	0.51	0.06–4.6	0.55			> 0.05
fT3 concentration at presentation	0.93	0.53–1.63	0.8			> 0.05
Iodine uptake [%]	0.98	0.92–1.04	0.47			> 0.05

**Table 4:** Factors contributing to achieving euthyreosis in patients with TMNG treated with RAI

	Univariate logistic regression analysis			Multivariate logistic regression		
	Odds ratio	95% OR confidence interval	*p*-value	Odds ratio	95% OR confidence interval	*p*-value
Age [years]	1.04	0.99–1.08	0.056	1.04	1–1.09	0.048
Gender [men]	1.44	0.58–3.59	0.43			> 0.05
Thyroid volume [ml]	1.03	1.002–1.05	0.037	1.038	1.009–1.068	0.009
Tyrostatic drugs taken 2 weeks before therapy	0.767	0.3–1.92	0.57			> 0.05
fT3 concentration at presentation	1.22	0.79–1.89	0.34			> 0.05
Iodine uptake [%]	0.96	0.93–0.99	0.03	0.95	0.9–0.99	0.02

Moreover, we performed Cox proportional hazards model analysis for determining predictors of normal hormonal function after 12 months of observation (Tables 5 and 6). It has shown age, thyroid volume and radioiodine uptake to be statistically significant, however, only in the overall patients’ population.

**Table 5:** Cox proportional hazards model analysis – predictors of eythyreoidism after 12-months observation in all the patients

Predictor	*p*-value	Hazard ratio (HR)	95% HR CI
Age [years]	0.003	1.039	1.013 – 1.066
Thyroid volume [ml]	0.01	1.016	1.004 – 1.029
Iodine uptake [%]	0.03	0.968	0.94 – 0.99
fT3 concentration at presentation	0.83	1.023	0.83 – 1.26
Gender	0.89	0.953	0.49 – 1.86
Tyrostatic drugs taken 2 weeks before therapy	0.38	0.703	0.32 – 1.54

*CI* confidence interval

**Table 6:** Cox proportional hazards model analysis – predictors of euthyreoidism after 12-months observation in GD and TMNG patients

Predictor	Graves’ disease patients			TMNG patients		
	*p*-value	HR	95% HR CI	*p*-value	HR	95% HR CI
Age [years]	0.102	1.05	1.013 – 1.066	0.195	1.02	0.98–1.05
Thyroid volume [ml]	0.19	1.025	1.004 – 1.029	0.174	1.01	0.99–1.02
Iodine uptake [%]	0.49	0.975	0.94 – 0.99	0.229	0.97	0.94–1.01
fT3 concentration at presentation	0.84	1.051	0.83 – 1.26	0.947	0.99	0.78–1.26
Gender	0.6	1.75	0.49 – 1.86	0.734	0.88	0.42–1.83
Tyrostatic drugs taken 2 weeks before therapy	0.73	1.7	0.32 – 1.54	0.511	0.75	0.32–1.75

*HR* hazard ratio, *CI* confidence interval

ROC curves were also depicted for these predictors to verify their accuracy in predicting the chances of rendering a patient euthyroid (Table 7). With this method we can obtain cut-off values with maximal sensitivity and specificity. For age as a stimulant, the optimal cut-off value was 58 with sensitivity of 86.5% and specificity – 56.5% (AUC: 0.752, AUC CI: 0.627–0.83, *p* < 0.001). In case of the thyroid volume (also a stimulating factor) the sensitivity and specificity were 58 and 80% respectively (AUC: 0.732, AUC CI: 0.646–0.819, *p* < 0.001) for the size of 35 cm3. Iodine uptake level was treated as a negative predictor and its threshold was set as 31% with the sensitivity – 65.3% and specificity of 70.3% (AUC: 0.666, AUC CI: 0.574–0.759, *p* < 0.001). ROC curves have also been depicted for subgroups of patients with GD and TMNG – results are presented in Tables 8 and 9.

**Table 7:** Predictors of euthyreosis in all the patients and their cut-off values using ROC curves [*n* = 52]

Predictor	Cut-off value	AUC	95% AUC confidence interval	*p*-value	Sensitivity	Specificity
Age [years]	58	0.752	0.67–0.83	< 0.001	86.5%	56.5%
Thyroid volume [cm^3^]	35	0.732	0.646–0.819	< 0.001	58%	80%
Iodine uptake [%]^a^	31	0.666	0.574–0.759	< 0.001	65.3%	70.3%

*AUC* area under curve

^a^ Negative predictors

**Table 8:** Predictors of euthyreosis in patients with GD and their cut-off values using ROC curves [*n* = 8]

Predictor	Cut-off value	AUC	95% AUC confidence interval	*p*-value	Sensitivity	Specificity
Age [years]	64	0.75	0.5–0.97	0.02	62.5%	87.3%
Thyroid volume [cm^3^]	27.4	0.83	0.71–0.95	< 0.001	85.7%	79.6%
Iodine uptake [%]^a^	31	0.59	0.37–0.83	0.4	57.1%	77.8%

*AUC* area under curve

^a^ Negative predictors

**Table 9:** Predictors of euthyreosis in patients with TMNG and their cut-off values using ROC curves [*n* = 44]

Predictor	Cut-off value	AUC	95% AUC confidence interval	*p*-value	Sensitivity	Specificity
Age [years]	58	0.612	0.448–0.736	0.07	90.9%	27%
Thyroid volume [cm^3^]	41.04	0.632	0.509–0.756	0.03	48.8%	80.6%
Iodine uptake [%]^a^	31	0.615	0.49–0.741	0.07	66.7%	59.5%

*AUC* area under curve

^a^ Negative predictors

In contrast to above-mentioned findings, type of nodule did not vary significantly between the presented groups. Following factors were also verified regarding potential contribution to the outcome of therapy, using logistic regression analysis: gender, antithyroid drugs and beta-blockers’ usage before the treatment, thyroidectomy in the past, radioiodine distribution in the gland, administered dose and type of nodules. No statistically important correlation, however, was noted.

## Discussion

Treatment with radioiodine has been one of the most important therapeutic modalities in case of hyperthyroidism for many years [7]. Many physicians prefer to use large quantities of the isotope in order to achieve early hypothyroidism and avoid the necessity of administering another dose of ^131^I^−^. Prompting stability with hormonal supplementation in case of hypothyroid patients is a common clinical practice, however, makes the patient fully dependent on the medications. There have been studies aiming to depict an optimal dose of ^131^I^−^ that would maximize the chances of rendering a patient euthyroid, however, they did not take into consideration other factors predictive of such an outcome [22, 23]. In our study we have presented three such predictors: iodine uptake level, subjects’ age and the thyroid gland volume.

The statistical analysis showed that pre-therapeutic RAI uptake level correlated inversely with the chances of achieving euthyreosis in our patients. In further analysis, it has been shown, however, that it only occurs in case of TMNG patients and not in the GD group. This finding is partially in concordance with the study by M.A. Walter et al., where an inverse correlation was presented in both GD and TMNG patients [24]. There have also been studies proving low RAIU to contribute to a successful outcome of the therapy, meant by hypo- or euthyroidism, however, exclusively in GD [16, 17]. On the other hand, RAIU> 50% also has been shown to increase the incidence of hypothyroidism in case of patients with solitary pretoxic or toxic adenomas treated with radioiodine ^131^I^−^ [18]. Moreover, in patients with toxic goiter and high radioiodine uptake, RAI therapy resulted in a failure more frequently than in subjects with lower or moderate RAIU levels [25]. Authors suggested that this phenomenon could be attributed to the stunning effect, although normally such a situation occurs, when larger quantities of radioiodine are administered. It could be also explained by the fact that patients with larger RAIU levels could have a more active disease, which results in lower susceptibility to thyroid ablation with radioiodine. The reason for developing hyperthyroidism in patients with very high iodine uptake levels may be due to progressive destruction of the thyroid gland with subsequent release of the free hormones.

Etiology of the hyperthyroidism also contributes to the outcome of the therapy. We have shown that patients with TMNG have more predictors of achieving euthyroidism than patients with GD. Moreover, it seems that TMNG patients were more prone to achieving normal thyroid function than subjects in the GD group. This finding is in accord with observations made by other researchers [20]. It has also been shown that, conversely, in patients with GD hypothyroidism is a more frequent outcome of RAI therapy than in subjects with TMNG [12, 20]. The explanation of our finding could be that the extranodal tissue in TMNG exhibits less iodine uptake. As a consequence, it does not suffer as much damage as the nodules, which allows the gland to maintain its function after the therapy has ended [10]. In other words, ablated thyroid tissue in GD patients may not able to maintain its hormonal function after the radioiodine treatment.

In our study we also found that the more advanced age in the patients was a significant predictor of achieving euthyreosis. As other studies usually focus on depicting factors contributing to the success of RAI therapy (and not euthyreosis exclusively), we had little material to compare our results with [11, 12, 26–32]. Nonetheless, some papers have shown that younger patients were more likely to present persistent hyperthyroidism after RAI therapy [9, 18, 26, 33]. The reason behind it may be that younger thyroid tissue is more resistant to radiation than in older patients [18]. Similar observations were made by other researchers, who found early age of onset of hyperthyroidism to be a negative predictor of the treatment success [12, 34]. This, however, was strongly connected to the severity of hyperthyroidism, rather than with the young age itself. Schneider et al. also noted a positive correlation between the older age and the success of RAI treatment having performed univariate analysis, but after multivariate analysis patients’ age was no longer significant [27]. Moreover, there have also been studies proving younger individuals to have an increased risk of presenting hypothyroidism [19, 28, 29].

There are certain factors that, undoubtedly, have an effect on the outcome of ^131^I^−^ therapy, e.g. use of ATDs, corticosteroids, goiter size, severity of hyperthyroidism, low-iodine diet or smoking habits. We must not forget, however, that these modulators are influenced by age and an overall condition of the patient. Thus, the patient’s advanced age and the final result of treatment are linked, though in an indirect manner [35]. Our result could be attributed to the fact, that 84.6% of patients who responded positively to the treatment, were diagnosed with TMNG, which prevalence increases in elderly patients and is more frequent than GD [7].

We have also proven that individuals with larger volumes of thyroid were more prone to achieving euthyreosis than patients with smaller glands. In contrast, many studies showed an inverse correlation between thyroid gland size and a successful outcome of treatment (again hypo- or euthyroid) [12, 32, 33, 36–38]. On the other hand, no significant impact of the above-mentioned factor was noted in other researches or results turned out to be statistically insignificant after performing regression analysis [9, 11, 20, 27, 39, 40]. Differences between various papers may be explained by lack of an objective method of measuring thyroid gland size. Clinical examination or scintigraphy are less precise than ultrasound [41, 42]. Marković et al. suggested that an influence of the gland size on the outcome of therapy was present in case of normo-echogenic glands only [21]. Our data show that 22.6 cm3 was the mean volume for hypothyroid outcome group and is in concordance with results presented by Catarina Machado et al. (24.3 cm3) [43]. Similarly, mean thyroid volume of persistently hyperthyroid patients’ (54 cm3) was comparable with findings by Pfeilschifter et al., who observed failure of RAI therapy in individuals with thyroid’s volume larger than 50 cm3 [19].

Since the focus in this paper is on the predictors of achieving euthyreosis exclusively, the literature review was severely narrowed as the great number of the scientific papers depict factors contributing to overall success of the RAI therapy. It is, undoubtedly, one of the limitations of our discussion. We also need to mention that although a group of 144 patients is big enough to draw statistically significant conclusions, in order to make our findings more credible, a larger cohort should be examined – preferably in a prospective study. Moreover, it needs to be underlined that even after rendering a patient euthyroid, there is a low risk of converting to hypothyroid state in the subsequent years after therapy, especially in patients pretreated with ATDs, eventually requiring hormonal supplementation [44]. Lack of control group is another limitation.

As a follow-up to this research, we consider using the measurements of the effective thyroidal half-life and uptake of ^131^I^−^ as shown by Kobe C. et al. [45]. This approach has been shown to provide high therapy success rates in patients with GD [46]. It seems that this individual dosimetric approach not only gives a more precise data on the radiation exposure, but also may be more beneficial than the classical dosimetric methods.

## Conclusions

In summary, in our research we depicted predictors of achieving euthyreosis after treatment with a fixed dose of ^131^I^−^ in hyperthyroid patients. The more advanced age, larger volume of thyroid gland and lower iodine uptake values contribute to rendering the patient euthyroid. In our opinion, physicians should take these three factors into consideration, while discussing the treatment modality with the patient, especially in case of TMNG diagnosis. It seems that subjects with higher RAIU levels should require special attention, since high failure rates are prevalent among them. Perhaps larger doses of radioiodine or longer pre-treatment with ATDs to prompt stabilization could be beneficial in these cases. In patients with e.g. smaller thyroid volumes, individual dosimetric methods may be useful to maximize the chances of achieving therapeutic success. GD patients with mild intensity of the disease could benefit from a longer use of ATDs before transferring them for radioiodine treatment, as older age seems to contribute to rendering a normal thyroid function in these patients. Further studies on predictors of normal thyroid function in the individual dosimetric approach, would help elucidate, whether the same factors also play a role in achieving euthyroidism, using this method.

## Data Availability

The datasets used and/or analysed during the current study are available from the corresponding author on reasonable request.

## Abbreviations

ATDs: Antithyroid drugs
AUC: Area under cruve
CI: Confidence interval
GD: Graves’ disease
HR: Hazard ratio
OR: Odds ratio
RAIU: Radioiodine uptake
ROC: Receiver operating characteristic
(f)T3: (Free) triiodothyronine
(f)T4: (Free) thyroxine
TA: Toxic adenoma
TMNG: Toxic multinodular goiter
TSH: Thyroid-stimulating hormone
